# Phylogenomics and genetic analysis of solvent-producing *Clostridium* species

**DOI:** 10.1038/s41597-024-03210-6

**Published:** 2024-05-01

**Authors:** Rasmus O. Jensen, Frederik Schulz, Simon Roux, Dawn M. Klingeman, Wayne P. Mitchell, Daniel Udwary, Sarah Moraïs, Vinicio Reynoso, James Winkler, Shilpa Nagaraju, Sashini De Tissera, Nicole Shapiro, Natalia Ivanova, T. B. K. Reddy, Itzhak Mizrahi, Sagar M. Utturkar, Edward A. Bayer, Tanja Woyke, Nigel J. Mouncey, Michael C. Jewett, Séan D. Simpson, Michael Köpke, David T. Jones, Steven D. Brown

**Affiliations:** 1 LanzaTech Inc, Skokie, IL USA; 2 DOE Joint Genome Institute, Lawrence Berkeley National Laboratory, Berkeley, CA USA; 3 Oak Ridge National Laboratory, Oak Ridge, TN USA; 4 Department of Life Sciences, Ben-Gurion University of the Negev, Beer-Sheva, 84105 Israel; 5 Institute for Cancer Research, Purdue University, West Lafayette, IN USA; 6 Department of Biomolecular Sciences, The Weizmann Institute of Science, Rehovot, 7610001 Israel; 7 University of California Merced, Life and Environmental Sciences, Merced, CA USA; 8 Department of Bioengineering, Stanford University, Stanford, CA USA; 9 Department of Microbiology, University of Otago, Dunedin, New Zealand

**Keywords:** Metabolic engineering, Genomics

## Abstract

The genus *Clostridium* is a large and diverse group within the Bacillota (formerly Firmicutes), whose members can encode useful complex traits such as solvent production, gas-fermentation, and lignocellulose breakdown. We describe 270 genome sequences of solventogenic clostridia from a comprehensive industrial strain collection assembled by Professor David Jones that includes 194 *C. beijerinckii*, 57 *C. saccharobutylicum*, 4 *C. saccharoperbutylacetonicum*, 5 *C. butyricum*, 7 *C. acetobutylicum*, and 3 *C. tetanomorphum* genomes. We report methods, analyses and characterization for phylogeny, key attributes, core biosynthetic genes, secondary metabolites, plasmids, prophage/CRISPR diversity, cellulosomes and quorum sensing for the 6 species. The expanded genomic data described here will facilitate engineering of solvent-producing clostridia as well as non-model microorganisms with innately desirable traits. Sequences could be applied in conventional platform biocatalysts such as yeast or *Escherichia coli* for enhanced chemical production. Recently, gene sequences from this collection were used to engineer *Clostridium autoethanogenum*, a gas-fermenting autotrophic acetogen, for continuous acetone or isopropanol production, as well as butanol, butanoic acid, hexanol and hexanoic acid production.

## Introduction

Climate change due to the emission of greenhouse gases has resulted in increasing interest in the production of energy and chemicals from renewable resources^1^. Current production of liquid transportation fuels and chemicals relies almost exclusively on carbon-based products derived from fossil-based resources. There is a need to decarbonize the energy sector by developing and deploying technologies that enable sustainable energy and chemicals production.

In the past, ethanol, butanol, and acetone from microbial fermentation of plant biomass has produced chemicals and fuels from renewable raw materials. The clostridial acetone-butanol-ethanol (ABE) fermentation process is over one hundred years old^2^ and has been reviewed recently^3^. Briefly, during the first half of the last century the clostridial ABE fermentation was the second largest industrial fermentation process behind ethanol fermentation^4,5^. After World War II the production of solvents by the fermentation process reached a peak during the 1950s with plants in 11 countries in full production. During the 1960s the use of the fermentation route went into rapid decline due to the advances made in petrochemical technology that resulted in replacement by cheaper solvents produced from fossil based raw materials. By the end of the 1960s the ABE plants in the UK, Taiwan, Japan, and Puerto Rico had all closed. The last ABE plant in the US ended production in 1977 and the fermentation process was later phased out in of South Africa in 1983 and in Brazil in 1993. The fermentation process did continue to be used in the USSR, China, and Egypt for the strategic production of solvents, but these plants were also eventually closed. The historic industrial ABE fermentation was operated as a batch process that utilized different *Clostridium* species. Initially maize was used as the main raw material but the industrial process later switched to using cheaper molasses. Later semi-continuous cascade fermentation processes were developed and utilized in both Russia and China. Due to high cost of agriculture-based raw materials and low end-product titers the ABE fermentation process has remained at best marginally economically competitive^6^.

Previous studies have shown that the *Clostridium* genus is not monophyletic and has resulted in taxonomic reclassification of many species^3,7–9^. The genus *Clostridium* is a large and diverse group of Gram-positive, spore-forming, obligate anaerobes whose members can encode for traits such as highly efficient multienzyme self-assembled complexes called cellulosomes for renewable plant biomass (lignocellulose) breakdown, solvent production, gas fermentation, thermophily and pathogenicity^7,10^. Direct microbial lignocellulosic biomass deconstruction and fermentation to ethanol and butanol represent strategies for producing chemicals, although there remain challenges in engineering and deploying non-model microorganisms as robust commercial production platforms, as reviewed recently^11^. The development and application of various genetic tools and genome sequencing has enhanced the scope for the genetic modifications of *Clostridium* species. In particular, the genetic engineering for biobutanol production has enhanced the possibility of substantial breakthroughs in the future. A joint European project sequenced the genomes of 30 solvent-producing *Clostridium* species^8^. Solvent-producing species constitute two distinct phylogenetic clades and a broader phylogenomic analysis of the genus was constructed from additional genome assemblies in the GenBank database^7^.

A collaborative project between LanzaTech Inc., Oak Ridge National Laboratory (TN, USA), and the U.S. Department of Energy (DOE) Joint Genome Institute (JGI) sought to sequence up to 300 genomes of solvent-producing clostridia from the LanzaTech DJ (David Jones) strain collection using PacBio long-read technology (Award 10.46936/10.25585/60000855). The primary aim of the project was to increase the available genomic database to facilitate the mining of useful genomic sequences for potential application in biotechnology. The 270 sequences described and characterized here represent genomes from single colony isolates derived from a culture collection of industrial solvent-producing and reference clostridial strains assembled by Professor David T. Jones. This collection originated in 1980 at the University of Cape Town. The National Chemical Products (NCP) chemical and fermentation company that operated the industrial ABE fermentation process in South Africa from 1935 to 1983 funded a research group at the University to undertake research on the ABE fermentation process. The culture collection included NCP industrial production strains. When Professor David Jones moved from Cape Town to New Zealand, duplicates of the culture collection were transferred to the University of Otago. Subsequently, his research group undertook studies on the molecular taxonomy and phylogeny of the industrial solvent-producing clostridia^9,12^. To facilitate this, additional strains were donated by several international culture collections and when the NCP company was disestablished following a takeover, additional NCP production strains were added to the collection. LanzaTech acquired the culture collection from Professor Jones after he retired in 2007.

LanzaTech has been using the carbon-fixing chemolithoautotrophic *Clostridium autoethanogenum* to produce fuel ethanol in commercial scale continuous gas fermentations since 2018^13^. Solvent pathway gene sequences have been mined from DJ strains, screened, and incorporated into *C. autoethanogenum* to produce the non-native chemicals acetone and isopropanol in addition to ethanol from industrial gas streams^14^. An *in vitro* cell-free protein synthesis approach used gene sequences from this collection, in part, for the production of medium chain (C4-C6) fatty acids and alcohols^15,16^. Today, there is interest in engineering non-model microorganisms with complex or innately desirable traits (e.g. lignocellulose deconstruction), as well as moving valuable traits (e.g. production phenotype) into highly editable conventional platform biocatalysts such as yeast or *Escherichia coli*^17^.

In this study, we report on the expanded dataset of 270 genome sequences and analyse and characterize genome sequences, phylogeny, along with the content and diversity of key metabolic genes, prophage and CRISPR-systems, cellulosomes, and quorum sensing for solvent-producing clostridia as a resource for synthetic biology and strain development.

## Results

### Genome sequences, phylogenetic analysis and gene content diversity

The compendium of 270 genome sequences in our collection represents diverse isolates from around the globe and includes industrial production strains that date back to 1944. We provide important information linking genome sequences to relevant culture collection details and historical notes (Tables S1-3). The genome sequences generated and analysed as part of this study includes 194 *C. beijerinckii*, 57 *C. saccharobutylicum*, 7 *C. acetobutylicum*, 5 *C. butyricum*, 4 *C. saccharoperbutylacetonicum*, and 3 *C. tetanomorphum* strains. Key attributes and genome statistics are provided (Table S1). To facilitate tracking during genome sequencing, each isolate was assigned a specific David Jones (DJ) number. There are 53 DJ genomes representing international culture collection strains arranged phylogenetically (Table S2). The remaining 217 DJ strains are derived from the NCP industrial collection and belong to either *C. beijerinckii* or *C. saccharobutylicum*. The original NCP coding designations are provided along with Integrated Microbial Genomes (IMG) accession numbers (Table S3). Most genomes (207) consisted of nine or fewer contigs and were generated using PacBio single molecule sequencing technology, with 117 of these comprising a single contig and likely representing complete genomes. The remaining genomes, mostly generated using Illumina technology, had a median contig number of 78.

**Fig. 1:**
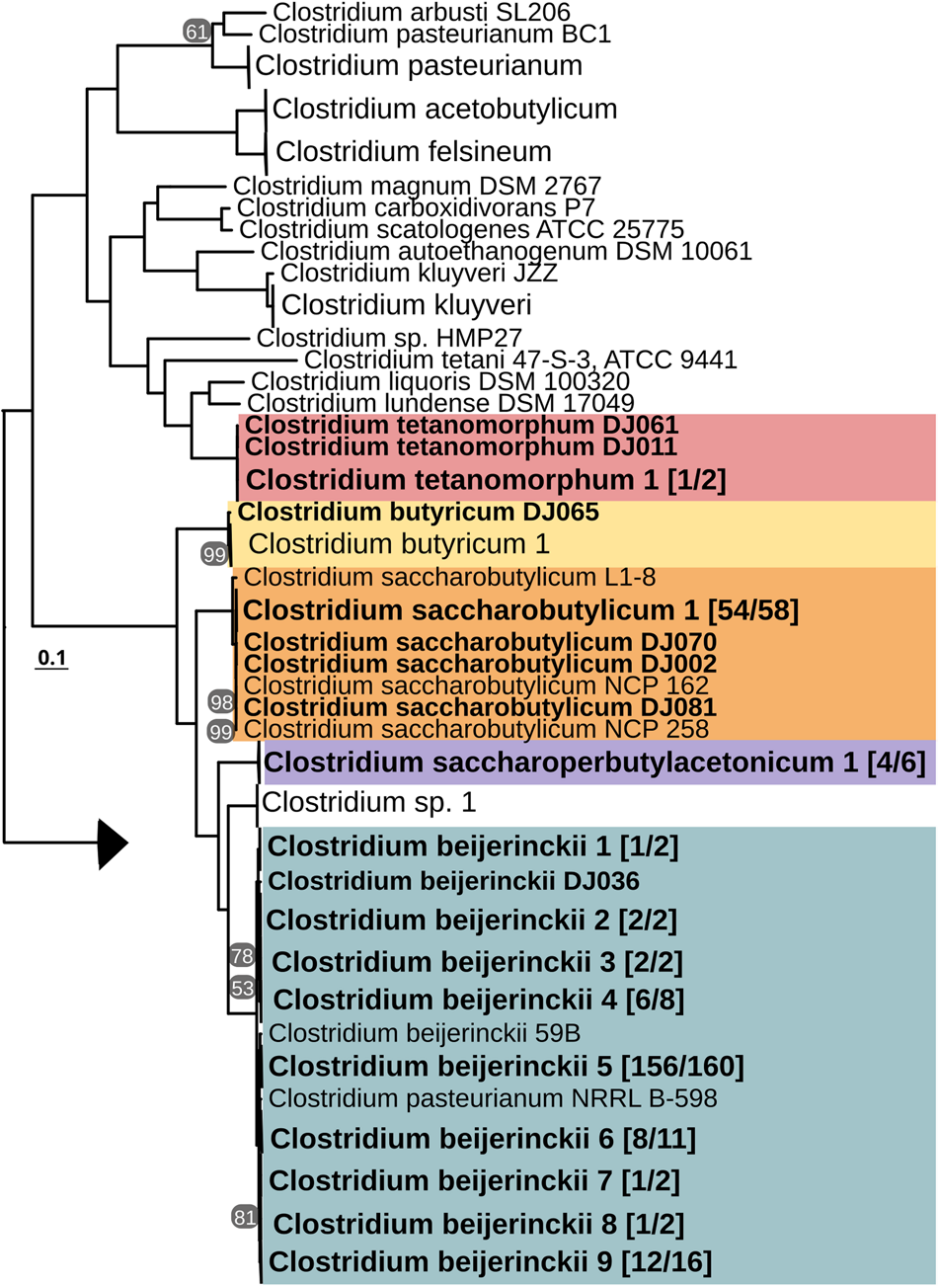
Phylogenetic based evolutionary history of DJ strains in the genus Clostridium. Species tree was constructed from a concatenated alignment of 175 single-copy panorthologs. Genomes are collapsed into clades if alignments are identical. Clades are highlighted in color if they contain strains from this study and numbers on the clades indicate the number of DJ genomes out of total genomes in the clade. All genomes in the tree that do not have the strain name suffix “DJ” are previously published reference genomes. Genomes that were created in this study have the DJ suffix in the strain name and are shown in bold, collapsed clades that contain DJ strains are shown in bold. *C. difficile* genomes are included as an outgroup, represented by a small arrowhead. Scale bar indicates substitutions per site. Bootstrap support values of below 100 are shown at the branches.

**Fig. 2:**
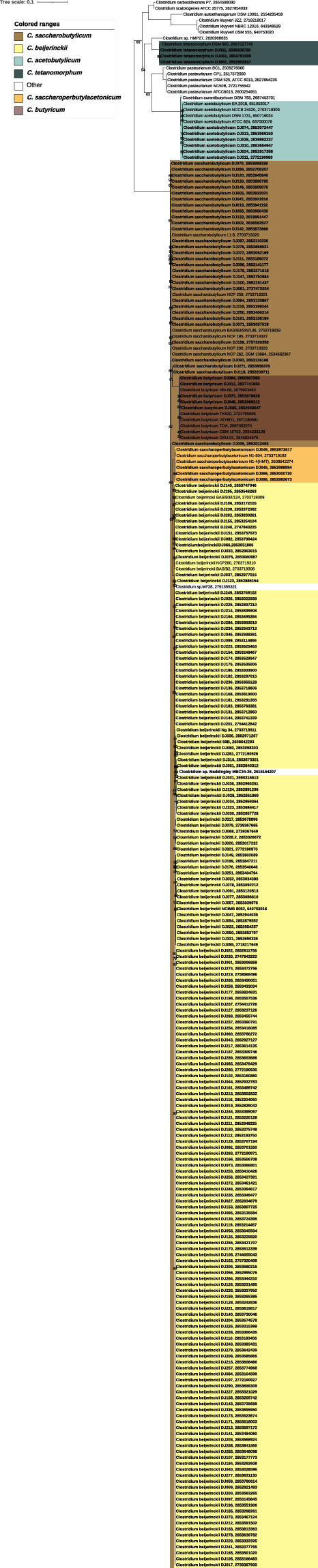
Evolutionary history of DJ strains in the genus *Clostridium* based on a concatenated alignment of 16S and 23S rRNA genes. In order to be retained in the dataset, genomes were required to retain the 16S rRNA gene with a length of at least 1,000 bp and the 23S rRNA gene with a length of at least 2,000 bp. Scale bar indicates substitutions per site.

**Fig. 3:**
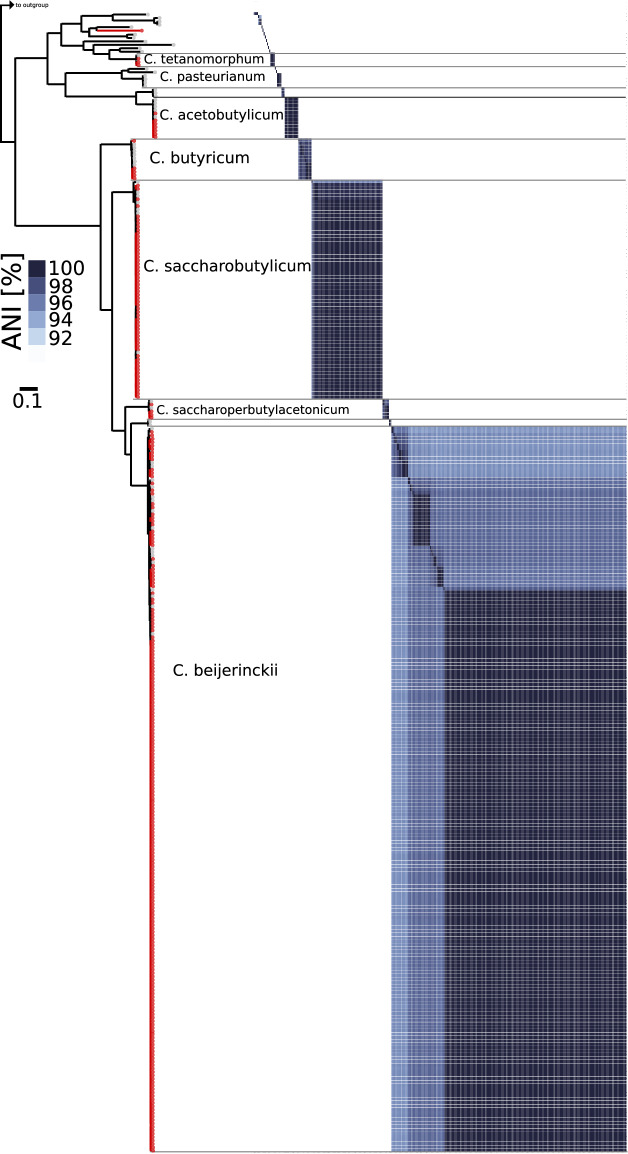
Phylogenomics based evolutionary history of the genus *Clostridium*. Maximum likelihood phylogenetic tree (LG4X + F) of the genus *Clostridium* after adding 270 genomes that were sequenced in this study (highlighted with red circles). Support values are indicated if below 100. Scale bar indicates the average number of substitutions per site. Pairwise average nucleotide identities (ANI) of 92% and above are displayed next to the phylogenetic tree. All major branches leading to species-level clades were fully supported (support value = 100), strain names and within species clade support values are shown in the detailed tree provided as Fig. 1.

Species were classified using a concatenation phylogeny of 175 *Clostridium* panorthologs including 16S and 23S rDNA genes (Fig. 1), 16S and 23S rDNA genes (Fig. 2), and using comparisons of genome-wide average nucleotide identities (ANI) (Fig. 3) as described previously^18^. Each of the major branches leading to species-level clades were fully supported, although as noted in previous studies misclassifications have been identified in the genus. Consistent with earlier findings^19^, the *C. diolis* group was found to be monophyletic with *C. beijerinckii* clades. The published *C. diolis* DSM 15410 and *C. beijernckii* VPI 5481 strains share an ANI of 98.08% (FastANI v1.3) and the former should therefore be reclassified as *C. beijerinckii*. Likewise, the ATCC17792 *C. kaneboi* strain should be reclassified as *C. acetobutylicum* and the ATCC 6013 *C. pasterurianum* strain should be reclassified as *C. beijerinckii*. In our phylogenetic analysis, the monophyly of *C. beijerinckii* DJ strains is supported and we kept the species designations as such rather than generating polyphyletic *C. beijerinckii* or *C. diolis* species and strains. *Clostridium pasteurianum* NRRL B-598 and *Clostridium* sp. MF28 also group with *C. beijerinckii* strains.

**Fig. 4:**
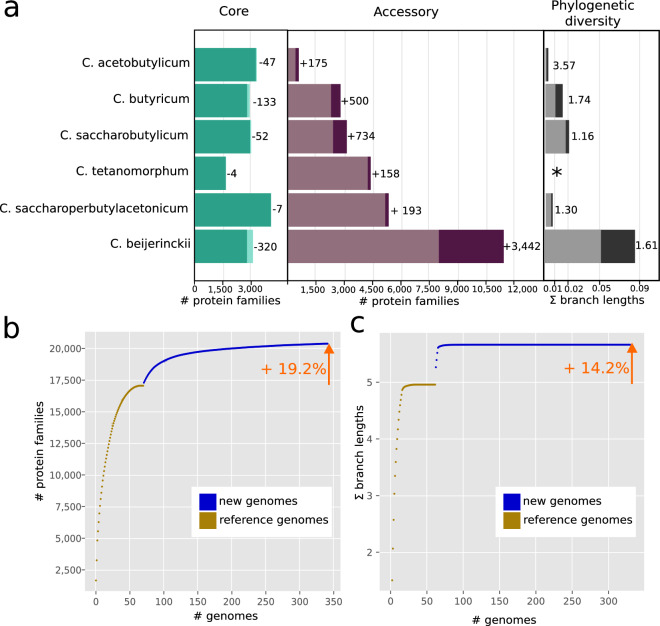
Expansion of protein families and phylogenetic diversity (PD) in the genus *Clostridium*. (**a**) Changes in the size of the core and accessory genome and PD (defined as the sum of branch lengths in a phylogenetic species tree) of solventogenic clostridia species after adding the newly sequenced *Clostridium* genomes. (**b)** Collector’s curve indicates the total increase in the number of protein families in the genus *Clostridium* before and after adding the new genomes. (**c**). Collector’s curve indicates the total increase in PD in the genus *Clostridium*.

To examine the expansion of protein families through the addition of new species and strains the sizes of core and accessory protein families were calculated before and after adding the genomes from this study. The pangenome size for each species increased, as did the phylogenetic diversity (Fig. 4A). A collector’s curves analysis showed a 19% increase in the number of protein families (Fig. 4B) and 14% greater phylogenetic diversity (Fig. 4C) across the genus after adding the newly sequenced *Clostridium* genomes. Most strikingly, the genomes generated in this study added more than 3,400 novel protein families within *C. beijerinckii* and increased phylogenetic diversity within this species. Similar to other studies, the addition of the first new genome with proteins without a paralog represents a new orthogroup, which leads to an immediate increase of the number of orthogroups, while with every additional genome the slope decreases as many proteins are added to already existing orthogroups in the plot.

### Core biosynthetic genes involved in solvent production

**Fig. 5:**
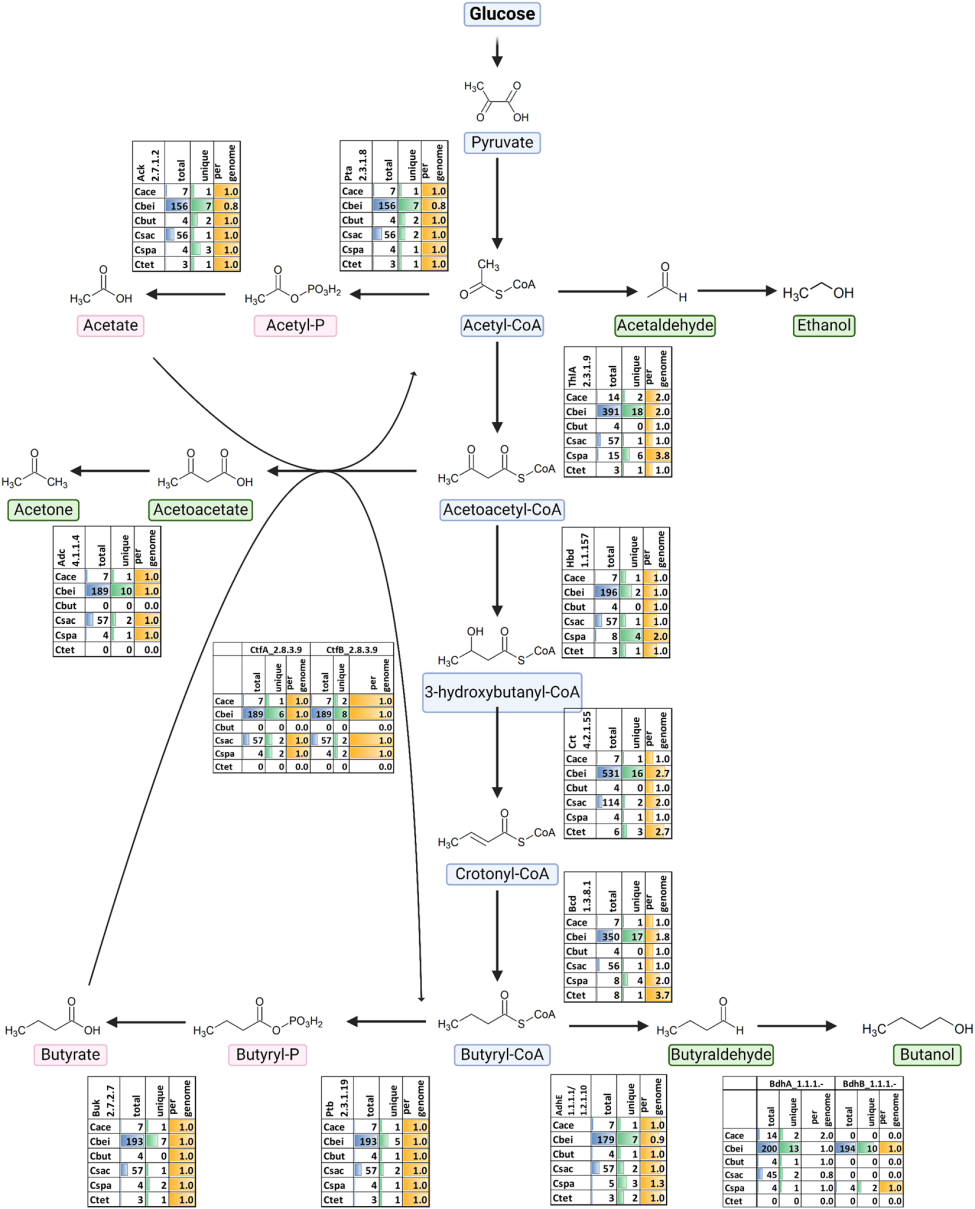
Gene count for genes involved in biosynthesis of ABE products. Total indicates the total number of genes for each genus found by homology search. Unique indicates the total number of sequences that differ by more than one amino acid found for each gene in the genus. Per genome is the number of sequences found per genus divided by the number of strains belonging to that genus.

Although the industrial solvent-producing clostridia fall into two different clades that do not share a close phylogenetic relationship, they do share the ability to produce solvents. Core biosynthetic enzymes involved in ABE production include thiolases (ThlA), 3-hydroxybutyryl-CoA dehydrogenase (Hbd), 3-hydroxybutyryl-CoA dehydratase (Crt), butyryl-CoA dehydrogenase (Bcd), phosphotransbutyrylase (Ptb), butyrate kinase (Buk), butanol dehydrogenase (Bdh) and aldehyde-alcohol dehydrogenase (AdhE2) for butyrate and butanol. ThlA, CoA-transferases A and B (CtfA, CtfB) and acetoacetate decarboxylase (Adc) are used for acetone production. Phosphate acetyltransferase (phosphotransacetylase, Pta), acetate kinase (Ack), and AdhE2 for acetate and ethanol have all been extensively studied^14,15^. Each clade differs significantly, based on the arrangement of the *sol* operon (*adhE-ctfAB-adc*) and the presence or absence of *rnf* and *pdc* genes. In this study, clade 1 is represented by *C. acetobutylicum*, and clade 2 by *C. beijerinckii*, *C. saccharobutylicum* and *C. saccharoperbutylacetonicu*m*. C. tetanomorphum* strains were included in the current analysis, although none of them belong to clades 1 or 2. There were 221 amino acid non-redundant sequences for core ABE genes within the collection and 48 sequences that were not in the NCBI database previously (in brackets), including 29(4) thiolases, 10(1) 3-hydroxybutyryl-CoA dehydrogenases, 24(8) 3-hydroxybutyryl-CoA dehydrogenases, 25(6) bifuricating butanoyl-CoA dehydrogenases, 17 (5) bifunctional aldehyde/alcohol dehydrogenases, 31(8) butanol dehydrogenases, 11(1) phosphate butyryl transferases, 12(1) butyrate kinases, 11(1) butyrate-acetoacetate CoA-transferase subunit A, 14(3) butyrate-acetoacetate CoA-transferase subunit B, 13(4) acetoacetate decarboxylase, 15(3) phosphate acetyltransferases and 16(3) acetate kinases (Fig. 5, Table S4). In the present analyses we define a unique sequence as having an amino acid (AA) sequence for a specific protein that has at least one AA differing from the other sequences in the protein group (several sequences with the exact same AA sequence represent one unique sequence). Key ABE biosynthetic protein sequences from type strains used to search DJ genomes are provided, along with strains where there were matches (Table S4). We observed an overall percentage of extracted unique sequences at approximately 6%, however for CtfAB, Adc and Ptb-Buk this was ~5% and ~3.5% for Hbd and Crt, which may indicate a higher degree of conservation for the latter. Several strains may have lost some ABE content as suggested by lower-than-expected gene counts per genome, which was observed for genes encoding ThlA and Crt and for genes where only one copy is normally present like Adc, CtfAB, Ptb-Buk, AdhE and Pta-Ack.

### Secondary metabolite analyses and quorum-sensing systems

Secondary metabolites have unique chemical properties that confer specific-bioactivity, therapeutic efficacy, and utility as ‘privileged’ chemical scaffolds in medicinal chemistry^20^. Widely found in plants, fungi and aerobic soil bacteria, novel secondary metabolic compounds have been discovered in anaerobic bacteria, including clostridia^21^, motivating our survey of the secondary metabolic potential of the collection. We chose an *in silico* approach, exploiting a growing collection of computational tools^22^, because many secondary biosynthetic gene clusters (BCGs) are silent during laboratory fermentation despite improved methods to detect their expression^23^.The secondary metabolic potential encoded in DJ collection was initially assessed using antiSMASH 5.0^24^, which identified 2,050 secondary metabolite biosynthetic gene clusters (BCGs) representing ten categories: type1 polyketide synthase (t1pks), non-ribosomal peptide synthase (nrps), nrps-like, bacteriocin, lantipeptide, lassopeptide, sactipeptide, pks-like, transAT-pks, and recorcinol. Every strain in the collection has at least one BGC. *C. beijerinckii*, the most numerous and phylogenetically diverse species, displays the greatest range of secondary metabolic potential, with 10/18 meta BGCs (mBGC) represented. The sactipeptide RiPP, which is present in every mBGC except group B, is a single, prevalent two gene BGC that encodes a SCIFF (Six Cysteine in Forty-Five residues) precursor peptide and its SAM dependent maturase. SCIFF derived peptides participate in quorum sensing and are nearly ubiquitous in clostridial genomes^25^, and, as expected, it occurs in each DJ strain apart from the *C. butyricum* group and in DJ046. We identified RRNPP-type quorum-sensing systems and accession numbers in current genomes are provided (Table S5).

**Fig. 6:**
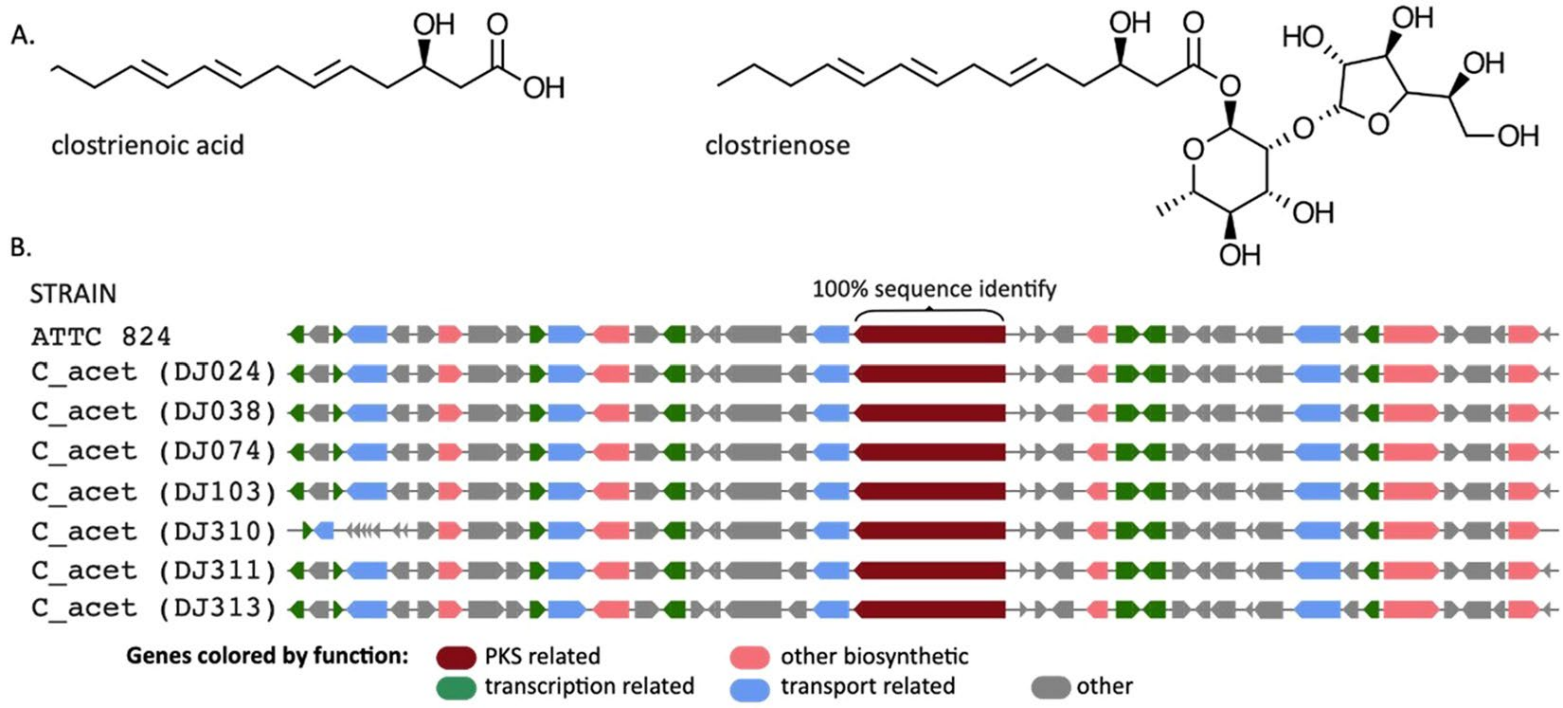
*C. acetobutylicum* type I PKS gene linked to clostrienic acid and clostrienose production is perfectly conserved in the seven DJ *C. acetobutylicum* strains. (**A)** Structures of clostrienic acid and clostrienose^26^. (**B)** Genome architecture of the type I PKS gene redrawn from antiSMASH 5.0 output. Note possible indication of recombination at 5′ end of DJ310.

**Fig. 7:**
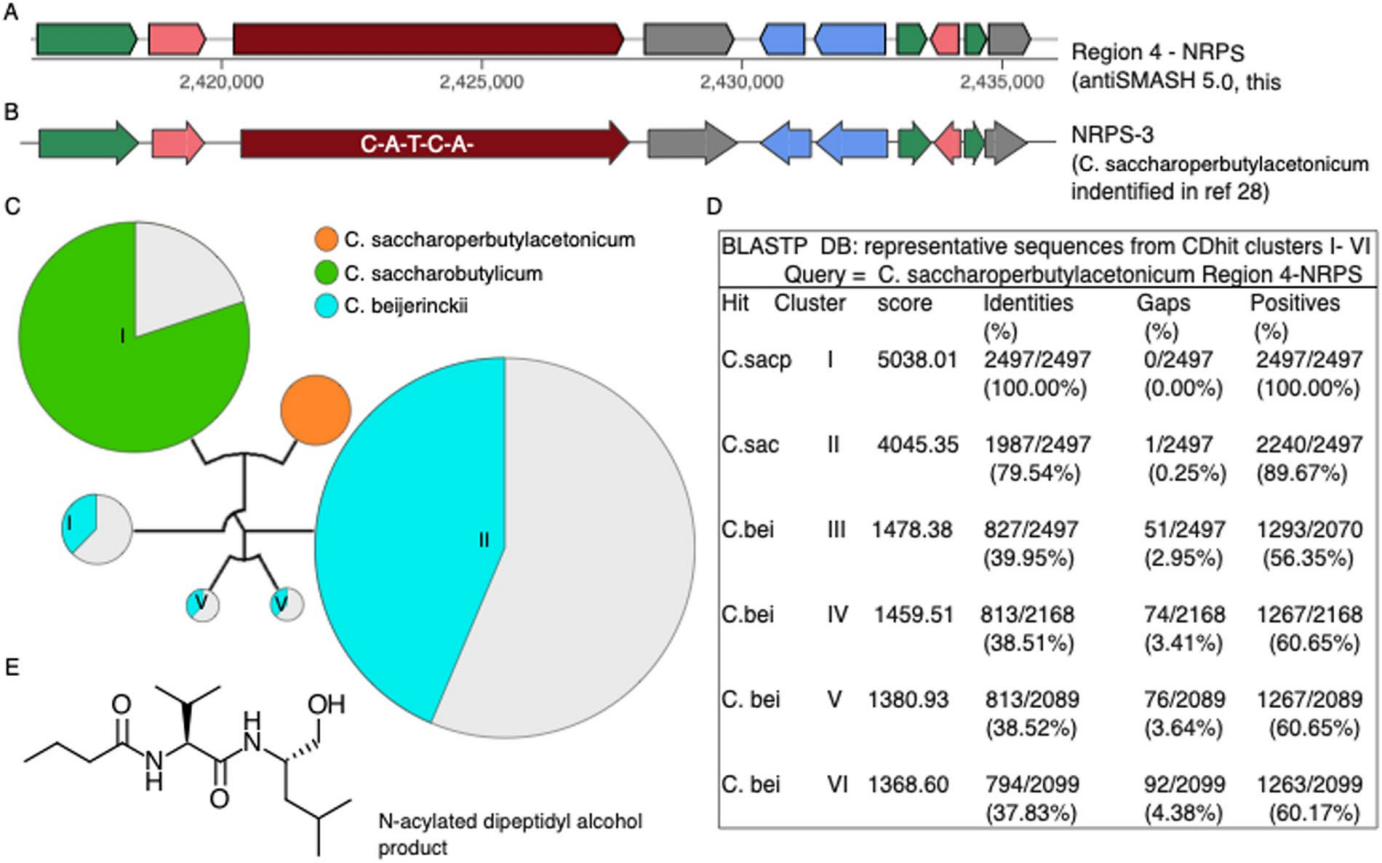
*C. saccharoperbutylacetonicum* NRPS gene linked to production of an N-acylated dipeptidyl alcohol is perfectly conserved in the four DJ *C. saccharoperbutylacetonicum* strains. (**A**) One of four NRPS BGCs identified by local antiSMASH 5.0 analysis of *C. saccharoperbutylacetonicum* N 1-4. (**B)** Cognate NRPS-3 BCG identified^28^. (**C)** CD-HIT clusters of BLASTP hits from DJ strains queried with Region 4 NRPS sequence; circle diameters proportional to number of cluster members. Tree is phylogram based on MUSCLE alignment of six representative cluster members. (**D)** Sequence similarity measured by probing one representative sequence from each CD-HIT cluster (total 6 sequences) using *C. saccharoperbutylacetonicum* representative as probe. (**E)** Structure of secondary metabolite product of this NRPS identified earlier^28^.

Herman *et al*. isolated clostrienoic acid and clostrienose from C*. acetobutylicum* type strain ATCC 824, characterized them by differential XCMS and NMR, and linked the biosynthesis of these compounds to a putative type I single module PKS (locus ca_c3355) by targeted, in-frame gene disruption, followed by comparative metabolomics^26^. The *C. acetobutylicum* ca_c3355 protein is perfectly conserved in the *C. acetobutylicum* DJ strains, as is the genomic context of the *pks* loci (Fig. 6), which is important because a single amino acid change can alter the product profile of these enzymes^27^. Li *et al*.^28^ identified a NRPS in *C. saccharoperbutylacetonicum* responsible for production of an N-acylated dipeptidyl alcohol, possibly related to butanol tolerance, which they characterized by comparative XCMS on wildtype versus knock-out strains, and by NMR analysis of purified compound. The NRPS is perfectly conserved in the four new *C. saccharoperbutylacetonicum* strains, is conserved in the new *C. saccharobutylicum* members, and partly conserved in some *C. beijerinckii* strains (Fig. 7). Clostyrylpyrones are a class of clostridial secondary metabolites recently discovered in *C. roseum*, where their biosynthesis has been mapped to the *csp* locus, and in particular to the CspD protein^29^. Although there are no *C. roseum* strains in the collection, BCGs undergo high rates of horizontal gene transfer^30^. We scanned the collection for clostyrylpyrone synthases, using the published *C. roseum cspD* sequence, and found no intact clostyrylpyrone synthase gene. However, a partial N-terminal *cspD* homolog is present. Recombination and rearrangement in the gene and in the surrounding locus appear to exemplify concerted, sub-cluster evolution prevalent in BGCs. Finally, Clostirubin, a polyphenolic polyketide antibiotic, has been isolated from *C. beijerinkii*, its structure elucidated^31^, and its total chemical synthesis reported^32^. Unfortunately, no sequence information or genetic information on the responsible gene(s) is publicly available, and we were unable to probe *C. beijerinckii* DJ strains for clostirubin synthases.

Additional analysis of BGCs and their families was conducted with *lsa*BGC using BGC prediction results from antiSMASH 7.0.0. AntiSMASH 7 yielded a total of 2850 BGCs, an increase of 800 over antiSMASH 5, largely due to its expansion of RiPP detection methods, though cyclic lactone autoinducers were also detected commonly throughout the genome collection. *lsa*BGC analysis using its GSeeF routine yielded 106 GCFs, a fairly large increase over the above BiG-SLICE results, owing to significant differences in the respective tools’ comparative routines. Comparison of BGC context across taxa was consistent with previous results showing the *C. beijerinckii* clade as having the most biosynthetic potential.

### Cellulosomal elements

**Fig. 8:**
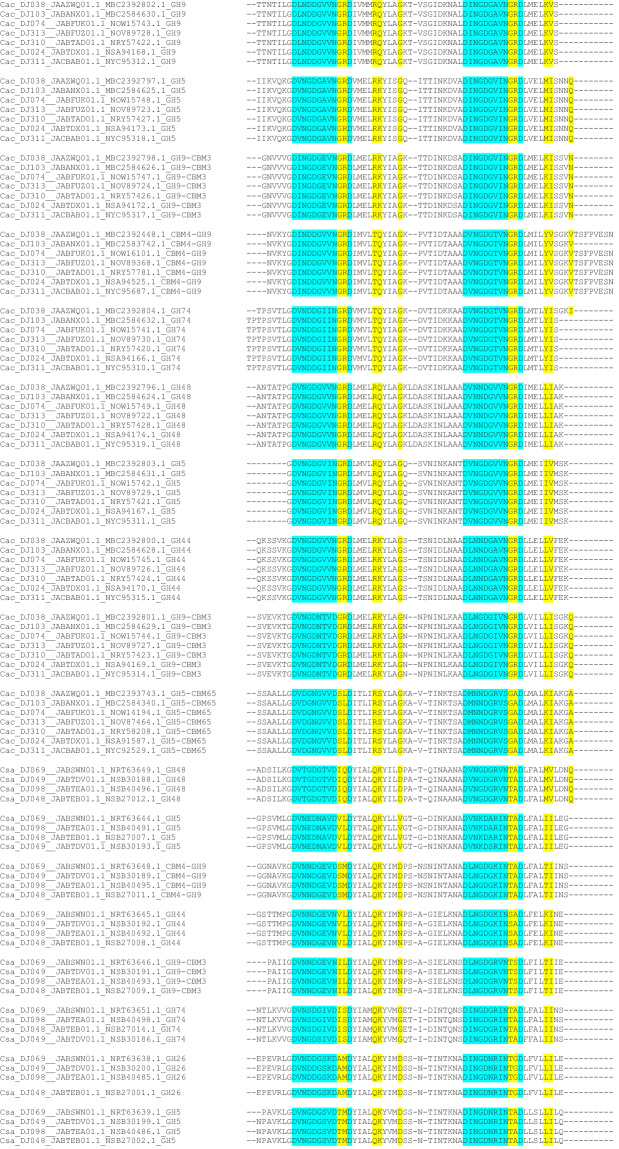
CLUSTAL O (1.2.4) multiple sequence alignment of dockerin modules from seven *Clostridium acetobutylicum* (Cac) strains and four *C. saccharoperbuytlacetonicum* (Csa) strains, examined in this study. Presumed recognition residues highlighted in yellow, calcium-binding motif in cyan.

**Fig. 9:**
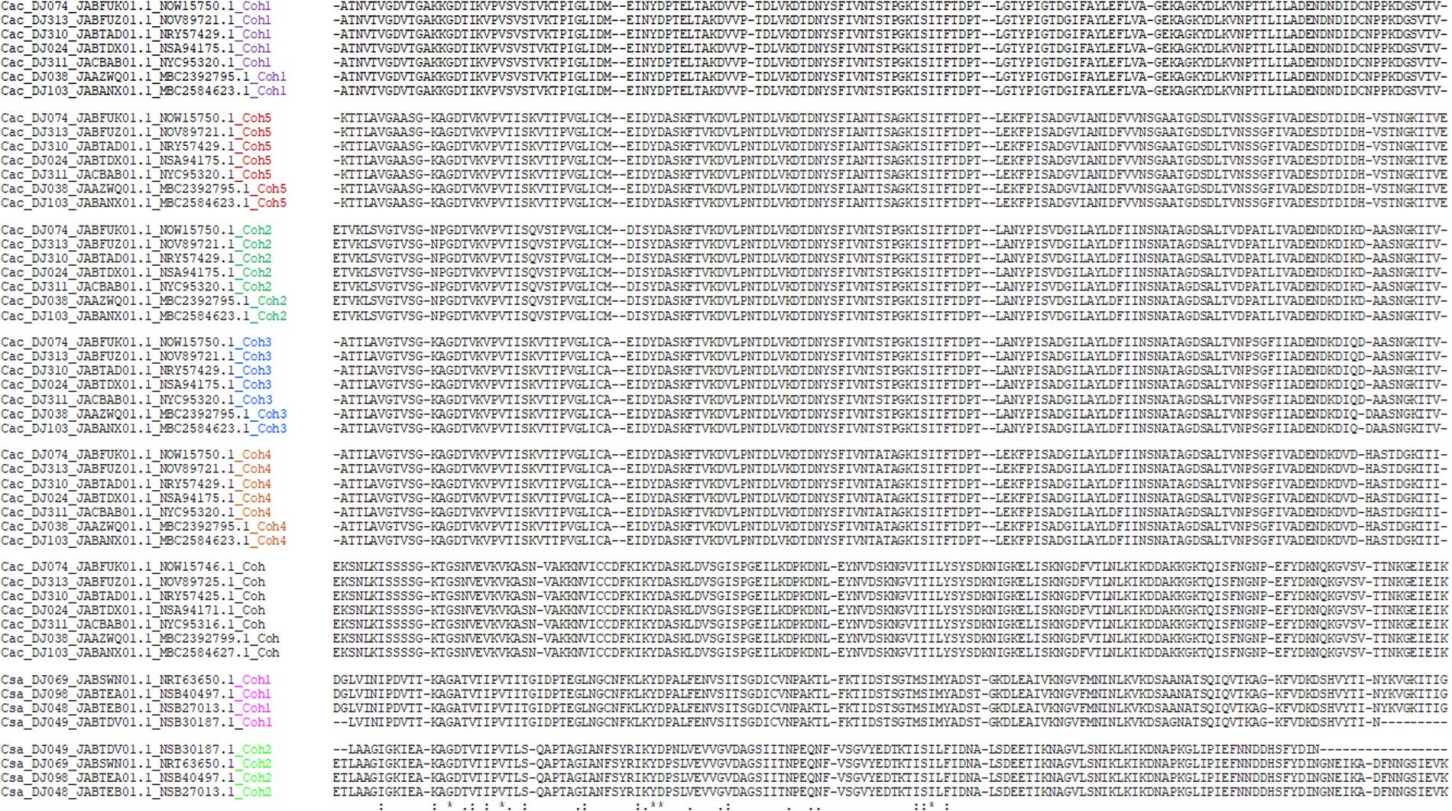
CLUSTAL O(1.2.4) multiple sequence alignment of cohesin modules. Seven *Clostridium acetobutylicum* (Cac) and four *C. saccharoperbuytlacetonicm* (Csa) strains from this study.

From the 270 genomes analysed, cellulosomal elements (multienzyme complexes for lignocellulose breakdown) were retrieved for *C. acetobutylicum*, and *C. saccharoperbutylacetonicum* (Table S6) and the distribution of glycoside hydrolases is reported (Table S7). Analysis of the seven *C. acetobutylicum* strains revealed that each genome contains 6 cohesins (5 in the scaffoldin sequence and one in the *orfX* gene) and 10 dockerins, as described previously for type strain ATCC 824. Similarly, the analysis of the four *C. saccharoperbutylacetonicum* genomes revealed a similar cellulosomal organization as in strain N1-4, including 2 cohesins and 8 dockerins. It is intriguing to note that the dockerin sequences of the given enzymes among the respective strains of *C. acetobutylicum* and *C. saccharoperbutylacetonicum* are exquisitely conserved—notably the predicted recognition residues (Fig. 8). Thus, the latter sequences of the GH48 dockerins from all the *C. acetobutylicum* strains are the same and different from those of the other enzymes (i.e., GH5, GH9, etc.), which are, among themselves, identical but different from those of the other enzymes. Likewise, the dockerin sequences (notably the proposed recognition sequences) are all essentially identical among all *C. saccharoperbutylacetonicum* strains for the specific enzymes (Fig. 8). Similarly, the scaffoldin sequences among the different scaffoldins are unvarying within the species, as reflected by the sequence identity of all their corresponding cohesin modules (Fig. 9).

### Prophage and plasmid diversity

**Fig. 10:**
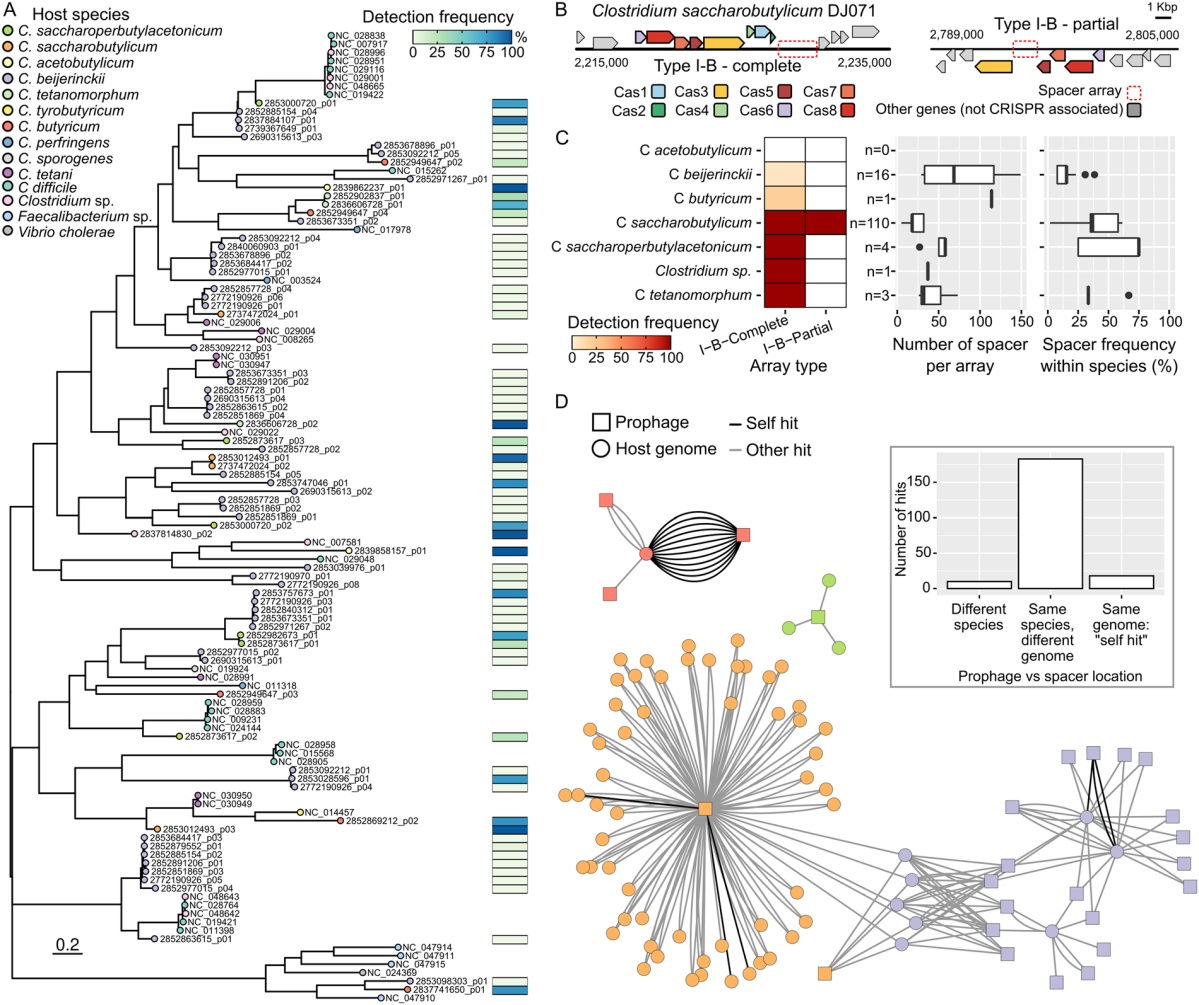
Diversity and interactions between predicted prophages and CRISPR-Cas systems across industrial clostridia. (**A**) Prophage CCP-like tree with prevalence across host species. The tree was based on a concatenated alignment following the CCP model^84^. (**B**) Schematic representation of the two types of CRISPR-Cas loci detected across industrial clostridia. Cas genes were annotated and colored as described earlier^34^. (**C**) Prevalence and spacer content of CRISPR arrays. For each species, the boxplots show the distribution of number of spacer for each array (middle panel) and the frequency detection of each spacer across CRISPR-encoding genomes from the same species (right panel). The number of CRISPR-encoding genomes is indicated to the left of the middle panel, and spacer detection frequency across the species were not included when only a single CRISPR-Cas genome was available (*C. butyricum*). (**D**) Global prophage:genome network. Genome nodes (circle) are connected to prophage nodes (squares) when a spacer from this genome matches the prophage with 0 or 1 mismatch. Genomes and prophages are colored based on the (host) species, edges are colored based on the prophage carriage of the genome: black for spacers matching a prophage from the same genome, gray for spacers matching a prophage from a different genome. The inset bar chart shows the distribution of connection type: matches to prophages from other species, matches to prophages from the same species but a different genome, and matches to prophages from the same genome, i.e. “self-hit”.

**Fig. 11:**
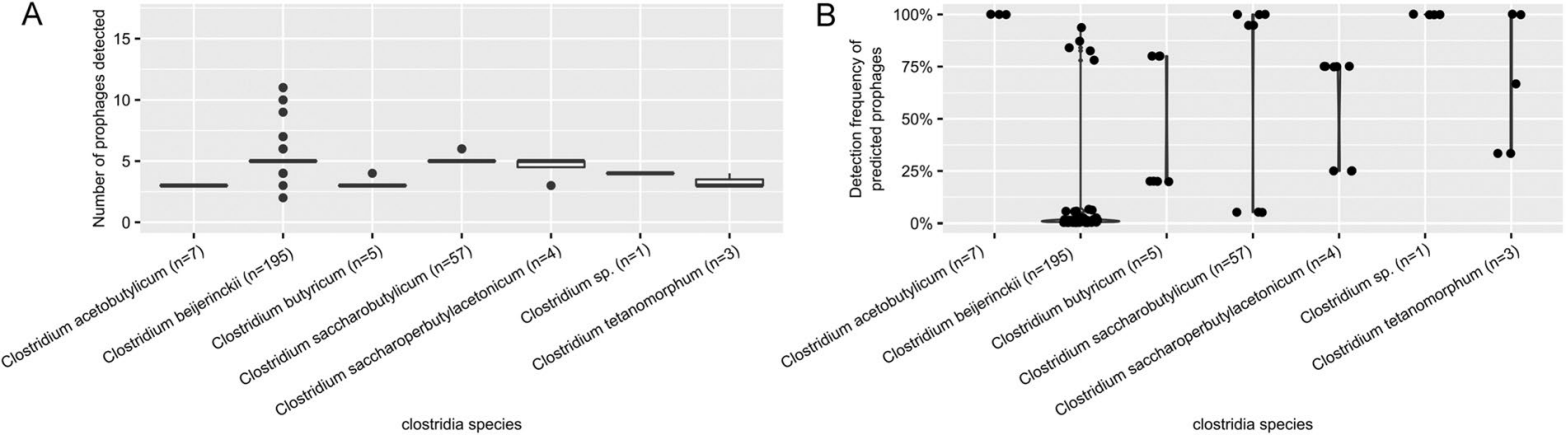
Number and prevalence of prophages across species and genomes. (**A**) Number of prophages detected by species, based on mapping host genome contigs to the non-redundant reference database of 100 industrial clostridia prophages. (**B)** Frequency of detection of each prophage across the members of each species. In both cases, a prophage was considered as detected if host genome contigs covered ≥80% of the prophage sequence at ≥80% nucleotide identity.

**Fig. 12:**
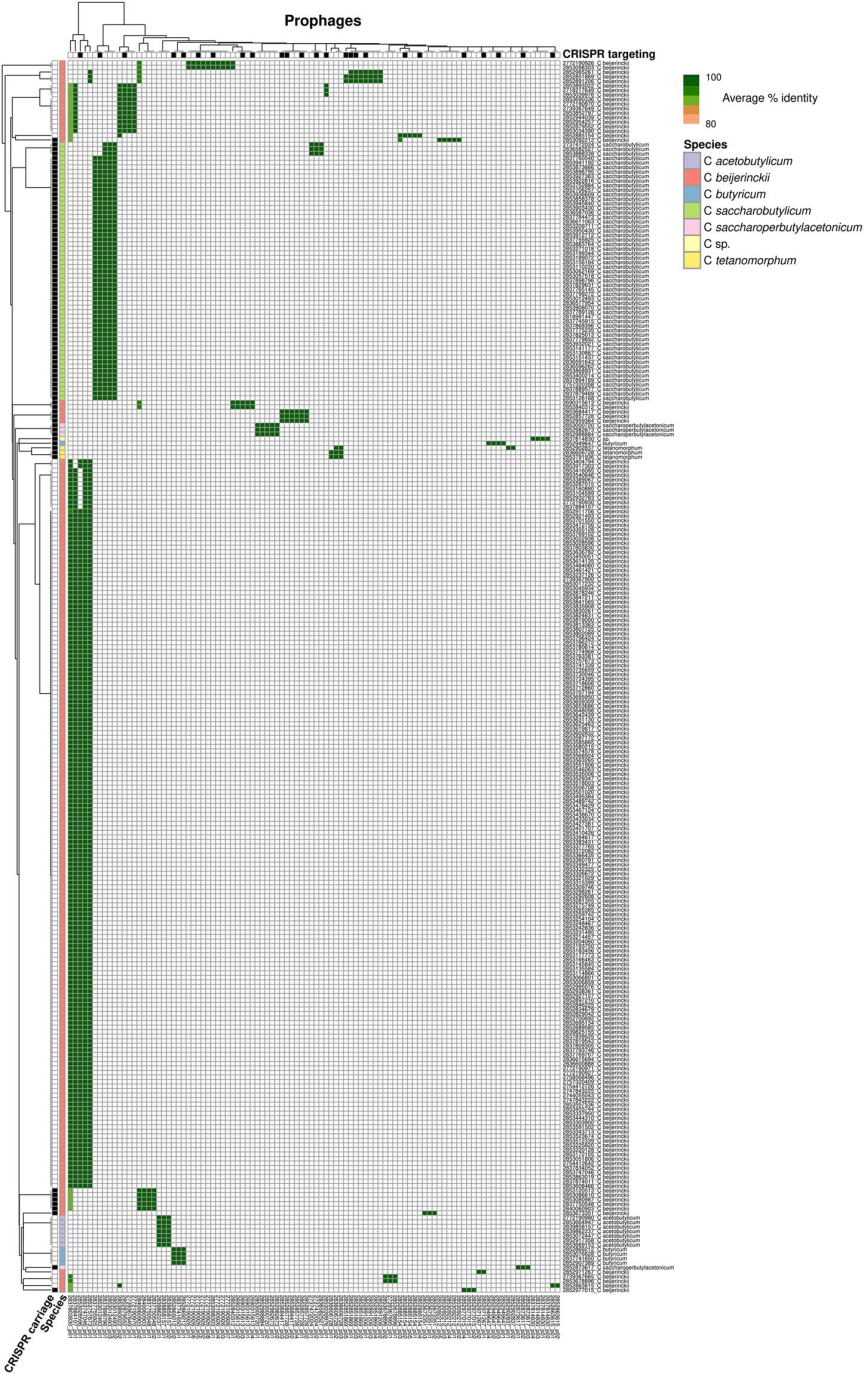
Distribution of prophages across of host genomes. The heatmap indicates the global average nucleotide identity percentage between a host genome contig and prophage representative sequence, for all cases where the host genome contig covered ≥80% of the prophage representative at ≥80% nucleotide identity. Both host genomes and prophages are automatically clustered based on these identity percentage values. Prophages targeted by at least one CRISPR spacer are highlighted with a black square. Host genomes encoding at least one CRISPR-Cas system are highlighted with a black square, and the species of the genome is indicated with colored squares.

Plasmid-like contigs were detected in 75 out of 270 genomes (Table S8); all *C. acetobutylicum* assemblies were predicted to contain 2.5 kb-11.1 kb plasmid sequences, as expected since key solvent pathway genes reside on a plasmid in the type strain^33^. Sensitivity and resistance to phage infection was of considerable importance in the industrial ABE fermentation process. To better understand the potential role of (pro)phages in the evolution of these *Clostridium* strains, prophages from different industrial species were identified and compared both to each other and with known references. A search of the 270 genomes using VirSorter identified 100 non-redundant (95% ANI; 85% AF) prophages. Overall, prophages predicted from the industrial clostridia genomes were either affiliated to the *Caudoviricetes* class, i.e. tailed phages (75%), or unclassified. Both concatenated phylogeny and gene-sharing network-based approaches suggested that these prophages were only distantly related to known reference phages from the NCBI Viral RefSeq database (Fig. 10). Specifically, only 2 prophages grouped with NCBI Viral RefSeq reference(s) in vContact2 genus-level clusters, while 34 belonged to clusters composed exclusively of clostridia prophages, and 64 were singletons (Table S9). The median number of prophages detected in each genome ranged from 3 to 5 depending on the host species, and most genomes included at least 3 detected prophages (Fig. 11). Notably, 3 genus-level clusters (VC_1_0, VC_5_0, and VC_21_0) included sequences from multiple clostridia species, suggesting a common evolutionary origin for some of these prophages. Prophages are found consistently associated with individual species of clostridia. In addition, each host species tends to be associated with unique sets of prophages: no prophages were shared between different host species, and 28 predicted prophages were detected in >65% of their respective host species member (Fig. 12). Combined, these data suggest a long-lasting and stable association of each host species with a distinct set of prophages.

### CRISPR-Cas diversity

As with most cellular organisms, industrial clostridia are constantly challenged by viruses and must maintain defence systems to counter these viral infections. CRISPR-Cas systems in the industrial clostridia genomes were therefore analysed for prevalence, diversity, and dynamics in closely related host species. CRISPR arrays were found to be unevenly distributed across industrial clostridia species. Searching for Cas genes and CRISPR-like repeat patterns uncovered 135 putative CRISPR arrays, which could be broadly classified into 2 types: a complete Type I-B CRISPR-Cas system, and a partial Type I-B CRISPR-Cas system which lacks genes associated with CRISPR spacer integration (Fig. 10B)^34^. The complete Type I-B system was the most common, detected in all the *C. saccharobutylicum*, *C. saccharoperbutylacetonicum*, and *C. tetanomorphum* genomes, and a minority of genomes from *C. butyricum* and *C. beijerinckii* (20% and 7%, respectively, Fig. 10C). Typically, only a single complete Type I-B operon was identified per genome, except in five *C. beijerinckii* genomes which encoded two distinct sets of Type I-B Cas genes, associated with two distinct spacer arrays with different repeats (Table S10). For these few instances of multiple copies of the Cas operon, it is possible that the Cas machinery may recognize different PAMs, enabling a more comprehensive defence mechanism. Meanwhile the partial Type I-B operon was only detected in *C. saccharobutylicum* genomes and found associated with spacers entirely different from the ones encoded in the complete Type I-B array. Since these partial Type I-B systems apparently lack the spacer acquisition module but use a repeat similar (though not identical) to the one associated with the complete Type I-B systems in the same genome, it is possible that these arrays are still active and leveraging the spacer acquisition machinery from the co-occurring complete Type I-B system.

While only encoded in a minority of genomes from this species, the CRISPR-Cas systems identified in *C. beijerinckii* were associated with a disproportionately high number of spacers, compared to other industrial clostridia genomes (Fig. 10C). Similarly, the overlap identified in terms of spacer sequences between genomes from the same species was typically lower for *C. beijerinckii*: while spacers were typically detected in >25% of the *C. saccharobutylicum*, *C saccharoperbutylacetonicum*, and *C. tetanomorphum* genomes, spacers were almost always detected in <25% of the genomes from *C. beijerinckii* (Fig. 10C). Finally, in the two species with varying presence of CRISPR-Cas systems (*C. beijerinckii* and *C. butyricum*), prophage carriage was clearly different between species members which encoded a CRISPR-Cas system and those which did not (Fig. 12). While these results could reflect to some level a sampling bias in this genome set, i.e. genomes within the *C. beijerinckii* species would be overall more diverse than the ones within other species, it does suggest that, at least based on this genome collection, *C. beijerinckii* CRISPR arrays are more active and dynamic than the one detected in most other industrial clostridia species.

Industrial clostridia CRISPR arrays defend against prophage from infecting neighbor species. We investigated potential associations between CRISPR spacers and phage genome sequences by comparing CRISPR spacers to 3 phage databases: NCBI RefSeq, which includes reference cultivated phages, IMG/VR v3, which is a collection of viral contigs identified in metagenomes, and the collection of industrial clostridia prophages established in this study (see above & Methods section). Using the NCBI RefSeq database, matches to two genomes were identified (*Clostridium* phage phiCT19406B and *Clostridium* phage phiCTC2B, Table S9). Considering that NCBI RefSeq includes 50 phages that infect members of the *Clostridium* genus also comprising pathogenic species such as *Clostridium difficile*, this confirms that phages infecting industrial clostridia described here are mostly distinct from previously isolated *Clostridium* phages. Conversely, a search of the IMG/VR v3 database uncovered 86 phage sequences with ≥1 spacer hits (Table S11). These sequences were obtained from human gut, soil/peat, and bioreactor samples, consistent with the known ecological distribution of clostridia species, and supports the contention that industrial clostridia CRISPR-Cas systems are actively used for defence against phages.

To further understand the activity of these CRISPR-Cas systems as anti-phage defence mechanisms, we next identified spacer hits to 25 distinct industrial clostridia prophages, and used these to build a prophage-spacer network (Fig. 10D). Remarkably, most hits (183 of 211) were between spacers and prophages found within the same species but in different genomes. This suggests that CRISPR-based phage defence is mostly used against phages infecting other members of the same species, but rarely against phages infecting other related species of clostridia. This could be due in part to other mechanisms preventing these phages to infect hosts across species including incompatibility in terms of virion attachment, replication or transcription machinery, or a lack of infection opportunity due to the species occupying distinct niches^35–37^. Within species however, spacer hits are mostly found to prophages not identified in the same genome, i.e. self-hits are very rare (Fig. 10D). Accordingly, most of these prophages (18 of 22) were only found in <10% of the genomes of the corresponding species (Table S10). This is consistent with a model in which CRISPR-Cas-based defence can limit the spread of prophages in new genomes but is ineffective once prophages have reached all or nearly all members of the population^38^.

## Discussion

No field of research has embraced and applied genomic technology more than the field of microbiology. During the past several decades, genomics-based approaches have had a profound impact on microbiology, our understanding of microbial species and the environment. Microbial genome sequencing projects have produced a wealth of new information and knowledge. The availability of numerous genome sequences and genomic databases have become an increasingly valuable resource to collect and disseminate the burgeoning amount of genomic data that has become available. Researchers can now extract important specific knowledge from various web-based, freely accessible genomic databases. The information from these genomic databases has the potential to enable comparative functional genome analysis as an effective approach for revealing the evolutionary relationships and provide novel sources of information for the understanding of the diverse metabolic capabilities and adaptation mechanisms of strains within the same and related species. They provide unique search, visualization, and analysis tools for organizing the large amounts of biological data currently available and make it easier for researchers to locate and utilize relevant information and facilitates an assessment of progress in functional genomics.

Microbial genomics has provided a foundation for a broad range of applications, from understanding basic biological processes, to a resource for genomics, genetics data and a repository for data mining. The availability of annotated genome sequences is central to genetic system development, not only to identify gene targets, and the diverse metabolic capabilities of strains for the engineering of microbes for industrial applications but also for supporting further specific study and research.

The aim of this project was to generate an expanded database to facilitate comparative functional genome analysis to explore the genetic and metabolic differences of the solvent-producing clostridia. The comparative analysis of over 300 genomes has demonstrated that the clostridial genomes are dynamic entities shaped by multiple factors and the functions of many previously uncharacterized features have been elucidated and tentative functional assignments have been made. This genome sequencing project, coupled with extensive metagenomic studies, is a resource that is expected to generate a more comprehensive picture of these important solvent-producing species.

The historic use of solventogenic clostridia to produce solvents acetone and butanol at industrial scales dates to the early 1900s^3^. However, the use of the industrial fermentation process was largely replaced by petrochemical alternatives in most countries during the second half of the last century. In an earlier study the genomes of 30 solventogenic *Clostridium* species from two distinct phylogenetic clades were sequenced^8^, and a phylogenomic analyses of the species was undertaken. A number of misclassified strains were identified that require taxonomic reclassification^39^, which is consistent with the findings of our study.

Most sequences reported in this study employed long-read sequencing technology. This has been shown to aid in resolving complex regions such as repetitive regions as in the case of multiple copies of rDNA operons resulting in high-quality genome sequences^40^. CheckM2^41^ quality analyses for genome completeness and contamination were completed for the 270 genomes. All had an estimated completeness of 100%, except strain DJ311 (99.75%) and 269 had estimated contamination less than 5%, with the DJ015 genome having an estimate of 10%. All results are provided together with genome stats and are consistent with the designation improved-high-quality draft (Table S1). In the present analysis we observed Illumina-derived genome assemblies were represented by higher numbers of contigs compared to PacBio-derived genomes, and consistent with earlier studies they contained fewer copies of rDNA operons overall. We did not observe obvious biases for metabolic gene contents between the sequencing technologies.

The expanded genomic database for solvent-producing clostridia generated by the joint LanzaTech JGI sequencing project can be accessed in GenBank. These 270 additional genomes are identified and coded as DJ genomes. The strains that were sequenced were selected from the DJ strain collection that consists of 53 examples originating from various international culture collections and 217 examples that originated from NCP industrial strain collection. These consist of 7 *C. acetobutylicum*, 194 *C. beijerinckii*, 5 *C. butyricum*, 57 *C. saccharobutylicum*, 4 *C. saccharoperbutylacetonicum*, and 3 C*. tetanomorphum* genomes. For convenience the genome sequences from the DJ collection were allocated arbitrary codes from DJ001 to DJ350. Conversion tables that list DJ genome designations, GenBank Accession numbers, JGI Integrated Microbial Genomes (IMG) numbers, along with the original strain names, codes, attributes, and historical annotations have been provided (Tables S1-3).

*Clostridium* is a large diverse genus of obligate anaerobes. Recently, Cruz-Morales *et al*. used genomic data from 779 strains to study the taxonomy and evolution of the group and showed clostridia are not a monophyletic group^7^. Their analysis confirmed that the group is composed of more than one genus and that the authentic *Clostridium* species are confined to what has been defined earlier as cluster I (*sensu stricto*) and that the *Clostridium* species belonging to this group can be divided into 2 major clades. Our analysis confirms this previously reported taxonomic classification that established that solvent-producing clostridia fit within these 2 different clades and are not closely related phylogenetically.

Solvent-producing species in Clade 1 include *C. acetobutylicum, C. aurantibutyricum*, *C. felsineum*, *C. roseum* and *C. pasteurianum*. The *C. acetobutylicum* strains were used for the commercial production of solvents from starch-based substrates and are characterized by having their solventogenic genes encoded on a plasmid. Although strains belonging to this species were isolated in Britain, North America, South America and Asia they all exhibit a very close genetic relationship. Despite being sub-cultured numerous times these genomes exhibit a remarkable degree of stability and conservation. The genome sequences include 3 strains of *Clostridium tetanomorphum*. This species is located in Clade 1 but it is not closely related to the other cluster of solvent-producing species. Although these bacteria are known to be able to produce low levels of butanol constitutively, they were never used for industrial purposes.

Solvent-producing species in Clade 2 that were used for the industrial production of solvents include *C. beijerinckii*, *C. saccharobutylicum* and *C. saccharoperbutylacetonicum*. These 3 species of industrial saccharolytic clostridia have their solventogenic genes encoded on the chromosome. These strains were used mainly for the commercial production of solvent from molasses and other sugar-based raw materials [substrates], although many of these strains are also able to produce solvents from starch, pentose sugars and other complex carbohydrates. Of the genomes sequenced, 194 strains are classified as *C. beijerinckii* and phylogenetic analysis indicates these strains belong to 4 different subgroups or subspecies. The *C. beijerinckii* subgroups are sufficiently different to likely warrant different subspecies or species designation. Group 1 strains include members known to produce isopropanol instead of acetone. While these strains were once used for commercial production many of the industrial strains have been lost over time. Group 2 strains contain many of the most well-documented and widely used industrial strains, that are predominately acetone producers. The Group 3 strains were mainly isolated due to their relevance in public health and food safety from various sources. These strains tend to produce low levels of solvents. Most of the industrial strains in Group 4 were isolated, patented and used by the Commercial Solvent Corporation (CSC) and provided to NCP, but were not deposited in international collections. Group 4 also includes several strains isolated in Japan in the 1950’s. The strains in the NCP collection holds unique value due to their documented history of propagation over a 40-year period. The most striking feature of these strains is their remarkable genetic stability.

The 57 *C. saccharobutylicum* genomes derived from strains in the NCP collection exhibit a very similar history and genetic characteristics. CSC filed US patents for two variants of this species designated gamma and delta. Although these genomes are closely related there is evidence that at least two subgroups of these strains can be identified. The *C. saccharoperbutylactonicum* strains were isolated and used in Japan as high butanol producers. The difference in the characteristics between the N1-4 strains and the N1-504 strain possibly qualifies them to be considered as different subspecies. Strains classified as *C. butyricum* constitute another large and ubiquitous species of saccharolytic clostridia belonging to this clade that do not produce solvents.

The taxonomic information generated from the expanded DJ genomic databases has enabled a comparative genome analysis that had revealed a greater understanding of the evolutionary relationships of strains within the same and related species and highlights the importance of phylogenomics for taxonomic studies. For example, over twice the number of *C. acetobutylicum* genome sequences are now available compared to when the study was initiated. Of the genomes sequenced for 194 strains classified as *C. beijerinckii* this study has clearly established these strains belong to 4 different subgroups or subspecies. The DJ genome sequences generated in this study have already been utilized in 3 recently published phylogenetic and taxonomic studies of solvent producing and *C. beijerinckii* species^7,39,42^. The *C. beijerinckii* genomes have provided a resource for comparison with newly isolated strains for 5 butyrate-producing strains from strong-flavor baijiu ecosystems^42^.

The number of new genomes for each species has been increased significantly and have expanded the number of core and accessory protein families. The expanded DJ genomic database has facilitated comparative functional analysis of the important metabolic differences of the solvent-producing clostridia. The solvent-producing species in Clade 1 are characterized by a common type I *sol* operon organization with a gene order *adhE–ctfA–ctfB*, with a separate *adc* operon located adjacent and being transcribed convergently. A *pdc* gene encoding pyruvate decarboxylase is present in these species, and *rnf* genes involved in the generation of an additional ion gradient from reduced ferredoxin are absent. The solvent producing members belonging in Clade 2 encode the *sol* operon in the gene order *ald–ctfA–ctfB–adc* and lack a *pdc* gene. Some species only produce acetone while other species have the capacity to further reduce acetone to isopropanol.

The expansion of available genomes and genes from solvent-producing clostridia has been exploited using *C. autoethanogenum* as a host organism^14,15^. Sequences for acetone and isopropanol biosynthesis were mined from this genome collection, screened using the largest autotroph library at the time and ultimately for continuous production at rates of up to ~3 g/L/h and ~90% selectivity^14^. Biosynthetic genes have also been mined for butanol, butanoic acid, hexanol and hexanoic acid production and the repertoire of genes offers similar possibilities for other host chasses^15,16^. The newly sequenced genomes could be further mined for different substrate transporters, such as xylose, sucrose or glycerol, other metabolic pathways such as lactate dehydrogenases (*ldh*), 1,3-propanediol oxidoreductases (encoded by *dhaT*) or glycerol dehydratases (*dhaBCE*), as described earlier^8^.

The expanded DJ genomic database has yielded significant new insights into the occurrence, diversity and distribution of genetic elements that encoded for a wide range of secondary metabolites and biosynthetic gene clusters (BGC) within this diverse group of solvent-producing clostridia. Notably, every genome in the DJ collection was found to encode for at least one BGC. Members of the 4 groups of *C. beijerinckii* were found to display the greatest range of secondary metabolic potential. If the inference is correct that Pks is solely responsible for synthesis of clostrienoic acid and clostrienose^26^, then *C. acetobutylicum* DJ strains are also likely producers although this requires confirmation. Other areas for potential follow up genetic and functional studies could include the bacteriocin lantibiotic group. The clostridia are also known to produce a wide array of other metabolites, including antibiotics such as chlorthiamide, a polythioamide product of *C. cellulolyticum* secondary metabolism that has activity against multi-resistant staphylococci. A recent analysis of the *C. beijerinckii* pangenome suggests that many of these novel properties may have gone unreported^39^.

From all the genomes analyzed, cellulosomal elements were only identified in *C. acetobutylicum* and *C. saccharoperbutylacetonicum*. Analysis of the *C. acetobutylicum* strains revealed that each genome contains 6 cohesins (5 in the scaffoldin sequence and one in the orfX gene) and 10 dockerins, as described previously for type strain ATCC 824^43^. Dockerin-containing proteins include the following glycoside hydrolase (GH) catalytic modules; three GH5s, four GH9s, one GH44, one GH48 and one GH74. Nine of these twelve cellulosomal genes are organized in a gene cluster, identical to that reported previously for *C. acetobutylicum* strains^43,44^. Similarly, the analysis of the four *C. saccharoperbutylacetonicum* genomes revealed a similar cellulosomal organization as in strain N1-4^45^, including 2 cohesins in a single scaffoldin and 8 dockerin-containing proteins. The annotation of the dockerin-containing enzymes is also analogous to those of the N1-4 strain, with two GH5s, two GH9s, one GH26, one GH44, one GH48 and one GH74. Moreover, five of the genes encoding the putative enzymes are organized in a gene cluster, together with the two-cohesin (bivalent) scaffoldin gene. The genome for DJ015 contained genes similar to *C. saccharoperbutylacetonicum* genes encoding ScaA and GH48 (99 and 100% sequence identities, respectively). However, a PCR assay was unable to confirm the presence of the genes for ScaA and GH48 in strain DJ015 (D. Klingeman, pers. comm). Together with the CheckM results, along with the highest number (665) of contigs (Illumina assembly) indicates this genome could be potentially excluded from future studies.

The investigation of the presence of prophages revealed that most genomes include at least three prophages and each host species tends to harbour a unique sets of resident prophages. This suggests a common evolutionary origin for most of these prophages within each species, indicating the possibility that many of these resident ages are inherited vertically, and have co-diverged along with their respective host species. As with many bacteria, the industrial species of clostridia all maintain CRISPR-Cas defence systems to counter viral infections. The CRISPR arrays were found to be unevenly distributed across the industrial clostridia species and the prevalence, diversity, and dynamics in closely related host species was found to vary quite widely. This study suggests that CRISPR-based phage defence is mostly used against phages infecting other members of the same species, but rarely against phages infecting other related species of clostridia. CRISPR-Cas systems can be further characterized *in silico*^46^ or using cell-free transcription-translation systems, as described for *E. coli*^47^. Information from the DJ genomic database has already been used in a recently published survey of resident prophages and R-type tailocins in the solvent-producing clostridia^3^.

In conclusion, this project has significantly advanced our understanding of this important group of industrial bacteria that will enhance their potential for future use and applications in biotechnology. There is a growing urgency to replace fossil-derived fuels and chemicals with more sustainable alternatives to mitigate carbon emissions. In addition to interest in the production of biobutanol as a chemical feedstock and biofuel^48^, there is a growing interest in other applications such as using clostridial-derived proteins as a potential alternative to animal protein^49^, as well as new process configurations utilizing designed microbial consortia^50^ and other considerations such as supply chain reassessments. This wealth of new information on *Clostridium* species, coupled with the repertoire of new genes for screening/testing in a range bacteria and yeast, will enable further functional and applied studies of this nature. The high-quality data, and analyses along with linkages provided here to international culture collections and historically important knowledge of industrial clostridia will facilitate future phylogenetic reclassifications, and further synthetic biology advances for novel strain construction.

## Methods

### Genome sequences

Genomes were sequenced at the JGI using Pacific Biosciences (PacBio) technology on either an RSII instrument (P6/C4 chemistry), or Sequel (v2.1 v2 or v3.0 chemistries) or using an Illumina NovaSeq (v1 chemistry) and have been reported previously^3^. All genome sequences were annotated using the JGI IMG pipeline, with the vast majority by version v.5.0.10 and details available for each at the JGI IMG database^51^. Completeness and contamination were estimated with CheckM2 (v1.0.2) with default settings^41^. Genomes used in this study are curated under the GOLD study ID Gs0118866, with links to raw sequence data available via JGI or via the NCBI SRA database. Contigs were classified into plasmid and chromosomal categories to determine plasmid presence or absence using PlasFlow v1.1.0^52^. For convenience, the 270 sub-projects are deposited under NCBI BioProject PRJNA990349. Strains analyzed in this study are shown Table S1, and include relevant culture collection details (Tables S2-3).

### Phylogenomics

Pairwise ANI was calculated with fastANI (v1.3)^53^ for 333 members of the genus Clostridia consisting of 61 previously published genomes available in the IMG/M database^51^ and 270 genomes sequenced in this study and 4 *Clostridioides difficile* genomes used as outgroup in the phylogenetic tree. Inference of clusters of COGs should be Clusters of Orthologous Groups (COGs) of proteins was performed with OrthoFinder (version 2.3.10)^54^ with default settings. A total of 192 single-copy panorthologs were selected as potential phylogenetic markers. For each of these panorthologs, protein alignments were built with MAFFT-linsi (version 7.294b)^55^ and phylogenetic trees constructed with FastTreeMP -lg (version 2.1.9 SSE3)^56^. Robinson-Foulds (RF) distances were then calculated between each possible pair of trees using ete3 (v3.0.0b35)^57^. The 17 most dissimilar trees (average RF distance >0.6) were then removed from the set of panorthologs. The remaining 175 single-copy panorthologs were used for phylogenomic analyses. The mafft-linsi alignments for the selected proteins were concatenated to a supermatrix. A maximum likelihood phylogeny was then inferred with IQ-tree (version 1.6.12)^58^ LG4X + F using the ultrafast Bootstrap Approximation^59^. The resulting phylogenetic tree was visualized in ete3^57^. To measure the increase in phylogenetic diversity (PD) after adding the newly sequenced *Clostridium* genomes, a phylogenetic tree was calculated as described above for each species in the genus *Clostridium*. The PD was then calculated as the difference of the sum of all branch lengths in the species-level trees with and without the newly sequenced genomes.

### 16S and 23S rDNA phylogeny

16S and 23S rRNA genes were identified with the Rfam^60^ models for the 16S rRNA gene (RF00177) and 23S rRNA gene (RF02641) using cmsearch (INFERNAL v1.1.1)^61^ on the same set of genomes that was used for phylogenomics. Only genomes were retained in the dataset that contained the 16S rRNA gene with a length of at least 1,000 bp and the 23S rRNA gene with a length of at least 2,000 bp. 16S and 23S rRNA genes were extracted, aligned with cmalign (INFERNAL v1.1.1)^61^ and concatenated. A phylogenetic tree was then inferred with IQ-tree (version 1.6.12)^58^ GTR + R10 using the ultrafast Bootstrap Approximation^59^ and visualized with ete3^57^.

### Central metabolism analysis

Core proteins for acid and solvent production were extracted based on amino acid sequence similarity to type strains for *C. acetobutylicum* ATCC824, *C. beijerinckii* NCIMB8052, *C. saccharoperbutylacetonicum* N1-4, *C. saccharobutylicum* DSM 13864 and *C. tetanomorphum* DSM665, which are provided along with DJ strains numbers that had a representative (Table S4). In addition, gene sets encoding Ptb-Buk, CtfAB-Adc and Pta-Ack had to have adjacent genes, and since there exist a multitude of short-chain acyl-CoA dehydrogenases often with undetermined specificities we examined sequences encoding Bcd with adjacent genes for EtfBA. In addition, ctfAB genes were required to be within 5 kb of genes encoding Adc to avoid other 3-oxo-transferases, and since there are many short-chain acyl-CoA dehydrogenases, often with undetermined specificities, we only extracted sequences for Bcd with adjacent EtfBA genes.

### Analysis of biosynthetic gene clusters for secondary metabolism

Biosynthetic Gene Clusters (BGCs) were identified in the genomes using local installations of antiSMASH 5.024^24^, 7.0.0, *lsa*BGC^62^ and BIG-SCAPE^63^ applying default parameters. Where a genome assembly was distributed across multiple contigs, prior to analysis, it was concatenated into a single fasta sequence, with a 10,000mer poly-G buffer inserted between each adjacent contig to prevent spurious colocalization of BGC motifs. BGCs were counted by parsing on the “product” line of the antiSMASH genbank output for each strain, and tabulated by BGC, meta-BGC (defined in results section), BIG-SCAPE class and clostridial species. Enzymes producing the known, structurally characterized, clostridial, secondary metabolites, were identified in the collection by Blastp implemented in a local instance of sequenceServer^64^, probing with published translated gene sequences. To segregate perfectly conserved from degenerate homologs hits were aligned with MUSCLE^65^, or clustered by percent identity using a local implementation of CD-HIT^66^. Pairwise global protein sequence alignments were built using the Needleman-Wunsch algorithm implemented in NEEDLE^67^.

### Identification of putative rrnp-type quorum-sensing system

Previously identified RRNP-type regulator gene sequences^68,69^ from *C. acetobutylicum* and *C. saccharoperbutylacetonicum* were used to perform a BLASTP search against all genomes in current study. We determined BLAST hits with >95% identity and 100% query coverage in all *C. acetobutylicum* and *C. saccharoperbutylacetonicum* strains in the current study except for the *C. saccharoperbutylacetonicum* strain DJ049 which has hits with >60% identity.

### Retrieval of cellulosomal elements in clostridia

Dockerin- and cohesin-containing sequences were retrieved from the predicted proteomes by local BLAST^70^, using known cohesin or dockerin sequences from the Bayer lab databases^71^. Hits below E-values of 10^−4^ above 45% of sequence identities and of lengths higher than 60 or 130 amino acids for dockerins or cohesins, respectively, were inspected individually for characteristic sequence features such as Ca^2+^-binding repeats and putative recognition residues of the dockerin modules^72,73^. Clustal Omega was used for multiple sequence alignments of dockerin modules^67^, and annotation of dockerin-containing genes was performed using dbcan2^74^. Annotation of glycoside hydrolases from was also performed with dbcan2.

### Prophage detection in industrial clostridia genomes

VirSorter v1.0.5^75^ was used to identify putative prophages in the clostridia genome collection (options: Virome database, predictions of category 1, 2, 4, and 5 selected). Prophage boundaries were further refined by (i) identifying canonical attachment sites as direct repeats of in a tRNA or upstream of an integrase gene within 10 kb of the original prophage prediction^75^, and (ii) using a “ping-pong blast” approach with all *Clostridium* genomes in the IMG database^51,76^. For each predicted prophage, predicted canonical attachment sites are considered first, then attachment sites detected via “ping-pong blast”, and the original coordinates are retained if neither approach identified potential new boundaries. Predicted prophages were next dereplicated at 95% ANI (average nucleotide identity) and 85% AF (aligned fraction) using MUMMER 4.0.0b2^77^ (options “–maxmatch–nooptimize”). The seed representative of each cluster was then cleaned using CheckV v0.7.0 to remove any remaining host region (option: “end_to_end”). Finally, all clostridia genome contigs were compared to this non-redundant prophage database using blastn v2.9.0^78^ (options “-task megablast -evalue 0.001 -perc_identity 70”, excluding hits <2,000 bp), and a prophage was considered as detected in a genome if it was covered by blastn hits on at least 80% of its length. Two approaches were used for taxonomic classification of prophages. A concatenated phylogeny based on known *Caudoviricetes* marker genes was built following the CCP77 guidelines^79^, with marker HMM profiles extracted from the VOGdb v97 (http://vogdb.org), multiple alignment computed with MAFFT v7.407 (option “einsi”), and tree built with IQ-Tree v1.5.5^58^ (option “-alrt 1000 -bb 1000”). Another classification was based on a gene-sharing network built with vContact 2_(v2021)^80^, including the 107 non-redundant prophages alongside 3,612 bacteriophage and archaeovirus genomes from NCBI Viral RefSeq v201^81^.

### CRISPR-Cas array detection

The detection of CRISPR-Cas arrays was done by the IMG annotation pipeline^51^, which uses a modified version of CRT^82^. Annotation of Cas gene clusters was then performed based on IMG functional annotation against the TIGRFAM database^83^ and each gene cluster was manually inspected to identify the type and completeness of the CRISPR array. CRISPR-Cas systems were then classified based on individual Cas gene annotation following the guidelines outlined earlier^34^. One array (2853080987_CRISPR_0) was on the edge of a contig, so that it was impossible to determine its completeness. Spacers were linked to individual arrays when detected within 10 kb of the Cas operon. To evaluate the diversity of spacers between clostridia genomes, spacer sequences were clustered at 100% nucleotide identity using cd-hit v4.8.1 (options “-c 1-d 0”^66^,). Spacers found in arrays that were associated with a Cas operon were matched to phage genomes in NCBI Viral RefSeq v201^84^, contigs from the IMG/VR v3 database^85^, and the prophage collection established in this study, using blastn v2.9.0+ with the following options optimized for short sequences: “-dust no -word_size 7”. Blast hits were then filtered to only retain hits showing 0 or 1 mismatch over the entire length of the spacer.

### Supplementary information

Supplementary Information

## Data Availability

Details of input data and genomes used in this study are referred to in the methods section above. JGI IMG web resources or data resources have been described^51,85^. Data underlying the phylogenomic analysis as well as the HMMs built from single copy panorthologs that were used in this study are available at https://github.com/NeLLi-team/djcollection. Output data are available in the Dryad open data publishing platform, under 10.5061/dryad.g4f4qrfx7^86^.

## Code availability

Software, versions and settings are described under appropriate methods subheadings, above.
